# Safety & efficacy of sodium glucose co-transport 2 (SGLT2) inhibitors in elderly patients with heart failure

**DOI:** 10.3389/fcvm.2026.1882576

**Published:** 2026-08-20

**Authors:** Anis Nabila Mohamad, Chandini Menon Premakumar, Abdullah Faiz Zaihan, Suharzelim Abu Bakar, Shairyzah Ahmad Hisham

**Affiliations:** 1 Department of Pharmacy, Hospital Jitra, Kedah, Malaysia; 2 Faculty of Pharmacy, University Kebangsaan Malaysia, Kuala Lumpur, Malaysia; 3 Department of Pharmacy, Hospital Shah Alam, Selangor, Malaysia; 4 Department of Pharmacy, Hospital Sultan Idris Shah, Serdang, Selangor, Malaysia; 5 School of Pharmacy, University of Nottingham Malaysia, Semenyih, Selangor, Malaysia

**Keywords:** acute kidney injury, elderly patient, LVEF, NYHA class, SGLT2 inhibitors, systolic BP ≤ 140 mmHg

## Abstract

**Introduction:**

Sodium-glucose cotransporter-2 (SGLT2) inhibitors have emerged as an important therapy in heart failure (HF). However, their safety and efficacy in elderly patients remain underexplored, as this group is often underrepresented in clinical trials.

**Aim:**

This study aimed to evaluate safety outcomes and describe changes in clinical parameters following initiation of SGLT2 inhibitors in elderly Malaysian patients with heart failure.

**Methods:**

A multicentre, single-arm, retrospective cohort study was conducted from January to December 2023 at two Malaysian hospitals. Patients aged ≥65 years initiated on empagliflozin or dapagliflozin were included. Safety outcomes assessed were acute kidney injury (AKI), orthostatic hypotension, genitourinary infections, hypoglycemia, volume depletion, and diabetic ketoacidosis (DKA). Efficacy outcomes included changes in left ventricular ejection fraction (LVEF), glycated haemoglobin (HbA1c), estimated glomerular filtration rate (eGFR), and New York Heart Association (NYHA) class. Descriptive statistics and chi-square tests were applied.

**Results:**

Among 126 patients (median age 70 years), AKI was the most frequent adverse event (12.1%), followed by orthostatic hypotension (3.0%) and urinary tract infection (1.5%). More than half (51.4%) showed LVEF improvement, while 38.2% of diabetic patients achieved better HbA1c control. Renal function improved or was preserved in 40.7%, and 21.4% demonstrated NYHA class improvement. Significant associations were observed between CKD and AKI (*p* = 0.003), and between systolic BP ≤140 mmHg and both eGFR improvement (*p* = 0.034) and NYHA class improvement (*p* = 0.029).

**Conclusion:**

In this retrospective cohort, SGLT2 inhibitor use was associated with observed improvements in selected cardiometabolic and functional parameters, with acute kidney injury being the most frequently recorded adverse event. However, given the single-arm retrospective design and the absence of a comparator group, these observed changes cannot be definitively attributed to SGLT2 inhibitor therapy alone and should therefore be interpreted as associations rather than evidence of a causal treatment effect. Careful clinical monitoring remains essential, particularly in patients with underlying renal impairment.

## Introduction

Heart failure (HF) is a complex and progressive clinical condition resulting from structural or functional abnormalities affecting ventricular filling or ejection. This ultimately leads to inadequate cardiac output, causing symptoms such as breathlessness, ankle swelling, and fatigue. It is often accompanied by signs including elevated jugular venous pressure, pulmonary crackles, and peripheral oedema (1).

Globally, HF affects approximately 56.2 million people, and its burden is expected to increase due to longer life expectancy and the rising prevalence of predisposing conditions. Economically, HF imposes a significant financial burden, mainly from recurring hospital stays and ongoing medication management (2, 3).

Age is known as a key predictor of the onset and progression of HF. It induces myocardial fibrosis, endothelial dysfunction, and diastolic impairment, further exacerbated by comorbidities such as hypertension, diabetes, and chronic kidney disease. Moreover, elderly patients with heart failure often face age-related syndromes like frailty, sarcopenia, cognitive decline, malnutrition, and polypharmacy, which are associated with reduced function and higher hospital admissions (4–6).

In Malaysia, HF prevalence rose from 669 to 809 per 100,000 between 1990 and 2019, with in-hospital, 30-day, and one-year mortality rates of 5.3%, 11.2%, and 33.1%, respectively (7). Projections indicate that Malaysia's elderly population is expected to increase from 8.1% in 2024 to 14.5% by 2040. This demographic is at increased cardiovascular risk, amplifying the incidence of HF. In 2023, HF-related care cost Malaysia USD 481.9 million, or 1.05% of national health expenditure, primarily driven by hospitalizations among elderly patients (2, 8, 9).

Guideline-directed medical therapy (GDMT) has improved HF management, particularly for heart failure with reduced ejection fraction (HFrEF), through agents like angiotensin-converting enzyme inhibitors (ACEIs), beta-blockers, mineralocorticoid receptor antagonists (MRAs), and SGLT2 inhibitors. However, therapeutic options for HF with preserved ejection fraction (HFpEF) remain limited, focusing on symptom relief and comorbidity control. SGLT2 inhibitors were initially developed as glucose-lowering agents for type 2 diabetes. Still, they have subsequently demonstrated significant cardiovascular benefits, including reduced mortality and hospitalizations among patients with HF, regardless of diabetic status (1, 9, 10).

Despite their therapeutic potential, elderly patients have been underrepresented in pivotal SGLT2 inhibitor trials, prompting concerns regarding the generalizability of the findings. Age-associated physiological alterations, multimorbidity, and polypharmacy may elevate the risk of adverse events including hypotension, volume depletion, urinary tract infections, and renal impairment (11, 12). In Malaysia, national guidelines endorse the use of SGLT2 inhibitors in HF; however, these recommendations are predominantly based on international data, with limited age-specific evidence. This situation creates uncertainty in clinical practice and may affect treatment outcomes in elderly patients (9).

This study aims to address the existing knowledge gap by describing the safety profile and changes in clinical parameters following SGLT2 inhibitor initiation in elderly patients with heart failure. The primary aim was to describe the safety outcomes and observe changes in LVEF, HbA1c, eGFR, and NYHA functional class over 12 months following SGLT2 inhibitor initiation. Ultimately, these findings may support safer, evidence-based clinical decision-making and reinforce adherence to established guidelines for this high-risk population.

## Materials and methods

This multicentre, retrospective cohort study was conducted at Hospital Sultan Idris Shah, Serdang, and Hospital Shah Alam, both of which are under the Ministry of Health, Malaysia. It included patients aged 65 years and older with a clinical or echocardiographic diagnosis of heart failure who were initiated on empagliflozin or dapagliflozin between January 1 and December 31, 2023. Data collection occurred from March to May 2025 via the electronic health information system (e-HIS).

The inclusion criteria consisted of individuals aged 65 years and above with a confirmed diagnosis of heart failure who had initiated SGLT2 inhibitors during the study period and had at least one clinical or laboratory follow-up assessment within 12 months after treatment initiation. Patients were excluded if they had incomplete clinical data or a baseline creatinine clearance below 20 mL/min. This exclusion is consistent with the eligibility criteria used in major clinical trials such as EMPEROR-Reduced and aligns with current prescribing guidelines (9, 10, 13). Eligible patients were identified through convenience sampling, resulting in a final cohort of 126 participants.

Safety and efficacy outcomes were evaluated using clinical, laboratory, and diagnostic information documented in the electronic health information system (e-HIS) up to 12 months after SGLT2 inhibitor initiation. Safety outcomes included acute kidney injury (AKI), orthostatic hypotension, volume depletion, genitourinary infections, hypoglycaemia, and diabetic ketoacidosis (DKA). AKI was defined according to the Kidney Disease: Improving Global Outcomes (KDIGO) criteria as an increase in serum creatinine of ≥0.3 mg/dL (26.5 μmol/L) within 48 h or ≥1.5 times the baseline value within 7 days (14). Hypoglycaemia was defined as a documented blood glucose level <3.9 mmol/L or a clinician-recorded diagnosis (15). Urinary tract infection (UTI) was defined as a documented episode of urinary tract infection in the medical records. Diabetic ketoacidosis (DKA) was identified from documented diagnoses supported by available laboratory findings, including positive ketones, serum bicarbonate <18 mmol/L, and arterial pH <7.3 where available (15). Volume depletion or orthostatic hypotension was defined as documented dehydration or a reduction in systolic blood pressure of ≥20 mmHg or diastolic blood pressure of ≥10 mmHg within 3 min of standing, where documented (16). Efficacy outcomes were predefined as an absolute increase in left ventricular ejection fraction (LVEF) of ≥5% from baseline (17), a reduction of at least one New York Heart Association (NYHA) functional class, a reduction in glycated haemoglobin (HbA1c) of at least 0.3% from baseline (18), and an increase in estimated glomerular filtration rate (eGFR) of 3–5 mL/min/1.73m^2^ from baseline at 12 months using the CKD-EPI equation (19). LVEF values were extracted from routine echocardiography reports documented in the electronic health information system (e-HIS).

Data were extracted using the Pharmacy Query tool and documented in a structured Excel form, which captured demographics, comorbidities, renal function (creatinine, eGFR), cardiac profile (LVEF, NYHA class), glycemic control (HbA1c), and adverse events. Missing data were handled using the pairwise deletion method, where each analysis included patients with available data for the specific variable or outcome. This approach accounts for the variation in denominators across efficacy parameters (*n* = 72 for LVEF, *n* = 68 for HbA1c, and *n* = 108 for eGFR), which occurred because specific laboratory tests or echocardiographic assessments were not uniformly performed or documented across the participating centers during standard retrospective clinical follow-ups. Multiple imputation was intentionally omitted to prevent the introduction of statistical artifact into unrecorded clinical evaluations.

Descriptive statistics were used to summarize baseline characteristics. The normality of continuous variables was assessed using the Shapiro–Wilk test. Continuous variables were reported as mean ± SD or median (interquartile range, IQR), while categorical variables were presented as frequencies and percentages. Associations between clinical variables and outcomes were analysed using the chi-square or Fisher's exact test, with *p* < 0.05 considered statistically significant. Due to the modest sample size (*n* = 126) and the low number of outcome events (e.g., *n* = 16 for AKI), multivariable logistic regression was intentionally omitted to avoid model overfitting and violation of the Events Per Variable (EPV) principle. Consequently, statistical analyses were restricted to exploratory univariate comparisons, and the findings should be interpreted as exploratory and hypothesis-generating. Ethical approval was granted by the National Medical Research Register (NMRR ID-25-00072-SMB [IIR]) and the Universiti Kebangsaan Malaysia Research Ethics Committee (JEP-2025-164).

## Results

A total of 126 elderly patients with heart failure who initiated SGLT2 inhibitor therapy were included in the analysis. The baseline median age was 70.0 years (IQR: 67.0–72.8), with a predominance of males (78.6%) and Malay ethnicity (61.9%). The majority had dyslipidemia (95.2%), followed by ischemic heart disease (84.1%), hypertension (83.3%), and diabetes mellitus (80.2%). Most patients were commenced on empagliflozin (96.0%), whereas a small proportion received dapagliflozin (4.0%) (Table 1).

**Table 1:** Demographic characteristics of elderly patients with heart failure.

Parameter		
Demographic	Median, (IQR)	*n* (%)
Age	70.0 (67.0–72.8)	
Sex, *n* (%)		
Male		99 (78.6)
Female		27 (21.4)
Ethnicity, *n* (%)		
Malay		78 (61.9)
Chinese		16 (12.7)
Indian		32 (25.4)
Comorbidities, *n* (%)		
Ischemic Heart Disease, IHD	106 (84.1)	
Diabetes Mellitus, DM	101 (80.2)	
Dyslipidemia	120 (95.2)	
Hypertension, HTN	105 (83.3)	
Chronic Kidney Disease, CKD	39 (31.0)	
Chronic Obstructive Pulmonary Disease/Asthma, COPD	9 (7.1)	
Functional Heart Failure, *n* (%)		
NYHA I	91 (72.2)	
NYHA II	31 (24.6)	
NYHA III	4 (3.2)	
SGLT2 Inhibitor, *n* (%)		
Empagliflozin 10 mg	50 (39.7)	
Empagliflozin 12.5 mg	29 (23.0)	
Empagliflozin 25 mg	42 (33.3)	
Dapagliflozin 10 mg	5 (4.0)	
Medications, *n* (%)		
RAAS Inhibitors		
ACEi	33 (26.2)	
ARB	32 (25.4)	
ARNi	36 (28.6)	
Beta blocker	97 (77.0)	
MRA	73 (57.9)	
Loop Diuretics	69 (54.8)	

The baseline clinical status revealed a median left ventricular ejection fraction (LVEF) of 37.0% (IQR: 30.0%–46.5%) and a median systolic blood pressure (SBP) of 138 mmHg (IQR: 121–154 mmHg). The median baseline eGFR was 65.9 mL/min/1.73m^2^ (IQR: 48.5–82.2), while the median HbA1c among diabetic patients was 7.10% (IQR: 6.30–8.55) (Table 2).

**Table 2:** Baseline clinical and laboratory parameters among elderly patients with heart failure receiving SGLT 2 inhibitors.

Parameter	Median, IQR
Serum Creatinine (mmol/L)	98.0 (82.5–121)
eGFR (mL/min/1.73m^2^)	65.9 (48.5–82.2)
Hba1c (%)	7.10 (6.30–8.55)
LVEF (%)	37.0 (30.0–46.5)
SBP (mmHg)	138.0 (121–154)
DBP (mmHg)	72.0 (66.0–80.0)

**Figure 1:**
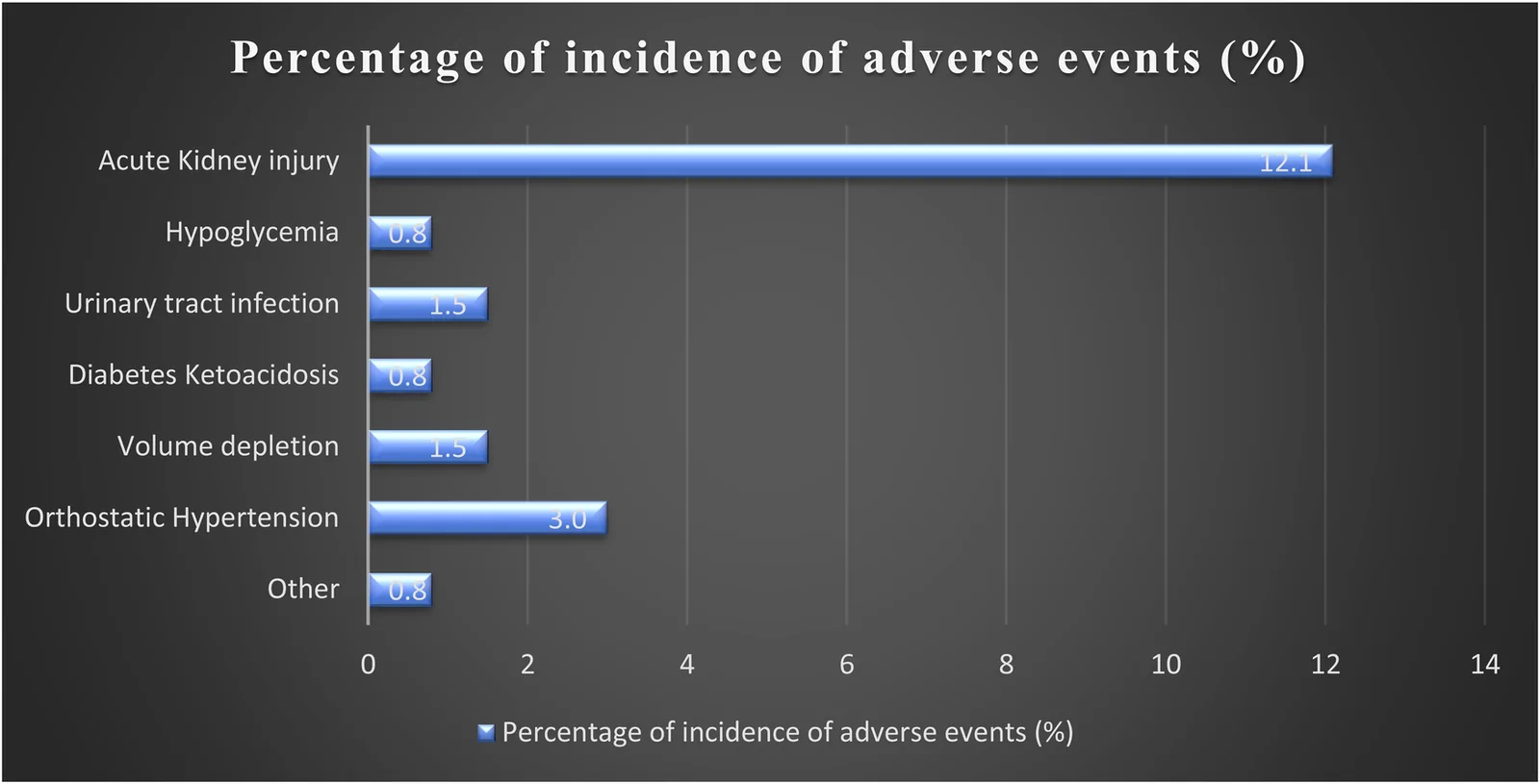
Percentage of incidence of adverse events among elderly heart failure patients receiving SGLT2 inhibitors.

Throughout the 12-month follow-up period, the safety profile of SGLT2 inhibitors remained acceptable. Acute kidney injury (AKI) was identified as the most frequent adverse event, affecting 16 patients (12.1%), followed by orthostatic hypotension in 4 patients (3.0%), volume depletion in 2 patients (1.5%), urinary tract infection in 2 patients (1.5%), hypoglycemia in 1 patient (0.8%), and diabetic ketoacidosis in 1 patient (0.8%). Figure 1 shows the temporal distribution of adverse events, highlighting the predominance of AKI throughout the study period.

**Figure 2:**
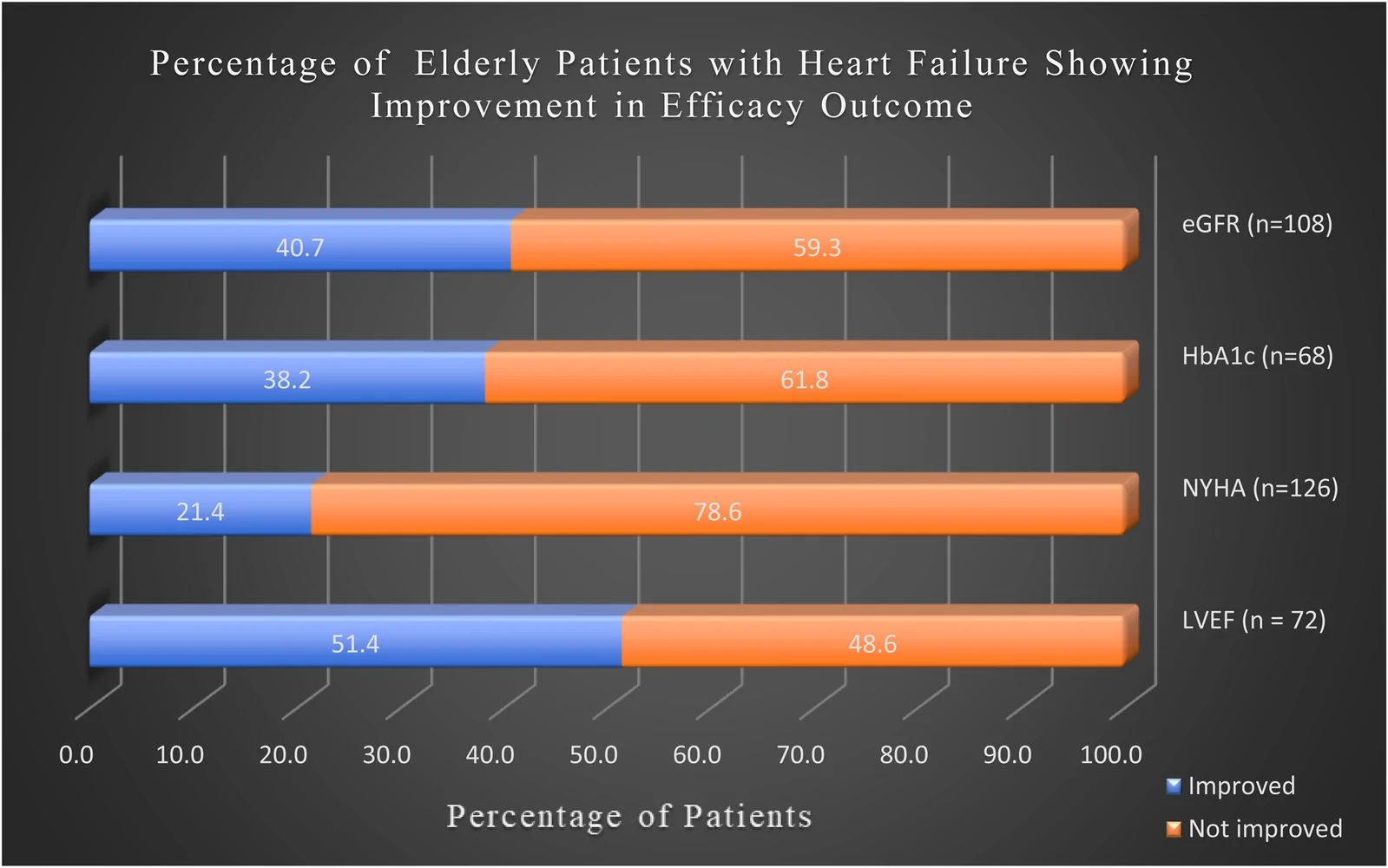
Percentage of elderly patients with heart failure showing improvement in efficacy outcome.

Regarding the efficacy outcomes, the available follow-up data indicated that 51.4% (*n* = 37/72) of patients experienced an improvement in LVEF, 38.2% (*n* = 26/68) of elderly patients with diabetes exhibited improvements in HbA1c levels; 40.7% (*n* = 44/108) showed enhancements in eGFR, and 21.4% (*n* = 27/126) demonstrated progress in NYHA class, as depicted in Figure 2. The variations in sample sizes across parameters reflect the retrospective nature of the study, where specific clinical evaluations were missing at follow-up and handled via pairwise deletion.

Statistically significant associations were observed between CKD and the incidence of AKI (*p* = 0.003), as shown in Table 3. Specifically, AKI developed in 25.6% (10/39) of patients with pre-existing CKD compared to only 6.9% (6/87) of patients without CKD. Moreover, a statistically significant association was found between SBP ≤140 mmHg and improvements in renal function (*p* = 0.034) (Table 4). Similarly, patients with SBP ≤140 mmHg were significantly more likely to show improvement in NYHA functional class (*p* = 0.029). No statistically significant associations were identified between age, diabetes status, or concurrent GDMT and any specific safety or efficacy outcomes.

**Table 3:** Association between patient-related factors on SGLT2 inhibitors and the incidence of AKI.

Variable	*N*	No AKI *n* (%)	AKI *n* (%)	*p* value
	126			
Demographics				
Age (years)				0.395^a^
≤70	72	63 (87.5)	9 (12.5)	
>70	54	47 (87.0)	7 (13.0)	
Comorbidities, *n* (%)				
Diabetes Mellitus, DM				0.145^a^
With Diabetes	101	86 (85.1)	15 (14.9)	
Without Diabetes	25	24 (96.0)	1 (4.0)	
Hypertension, HTN				0.632^a^
With hypertension	105	91 (86.7)	14 (13.3)	
Without hypertension	21	19 (90.5)	2 (9.5)	
Ischemic heart disease, IHD				0.693^a^
With IHD	106	92 (86.8)	14 (13.2)	
Without IHD	20	18 (90.0)	2 (10.0)	
Chronic Kidney Disease, CKD				0.003^a^^,^^*^
With CKD	39	29 (74.4)	10 (25.6)	
Without CKD	87	81 (93.1)	6 (6.9)	
Medications				
RAAS Inhibitors				
ACEi				0.469^a^
With ACEi	33	30 (90.9)	3 (9.1)	
Without ACEi	93	80 (86.0)	13 (14.0)	
ARB				0.205^a^
With ARB	32	30 (93.8)	2 (6.3)	
Without ARB	94	80 (85.1)	14 (14.9)	
ARNi				0.397^a^
With ARNi	36	30 (83.3)	6 (16.7)	
Without ARNi	90	80 (88.9)	10 (11.1)	
Mineralcorticosteroid antagonist, MRA				0.884^a^
With MRA	73	64 (87.7)	9 (12.3)	
Without MRA	53	46 (86.8)	7 (13.2)	
Loop Diuretics				0.344^a^
With Loop diuretic	69	62 (89.9)	7 (10.1)	
Without Loop diuretic	57	48 (84.2)	9 (15.8)	
SBP (mmHg)				0.256^a^
≤140	70	59 (84.3)	11 (15.7)	
>140	56	51 (91.1)	5 (8.9)	

* *p*-value of less than 0.05 is considered significant.

a Chi square test.

**Table 4:** Association between patient factors and eGFR improvement after initiation of SGLT2 inhibitors.

Variable	*n*	Improved eGFR *n* (%)	Not improved eGFR *n* (%)	*p* value
	108			
Demographics				
Age (years)				0.113^b^
≤70	61	27 (44.3)	34 (55.7)	
>70	47	17 (36.2)	30 (63.8)	
Comorbidities				
Diabetes Mellitus				0.206^a^
With Diabetes	87	38 (43.7)	49 (56.3)	
Without Diabetes	21	6 (28.6)	15 (71.4)	
Ischemic heart disease, IHD				0.265^a^
With IHD	91	35 (38.5)	56 (61.5)	
Without IHD	17	9 (52.9)	8 (47.1)	
Chronic Kidney Disease, CKD				0.053^a^
With CKD	33	18 (54.5)	15 (45.5)	
Without CKD	75	26 (34.7)	49 (65.3)	
Vital sign				
SBP (mmHg)				0.034^a^^,^^*^
≤140	63	31 (49.2)	32 (50.8)	
>140	45	13 (28.9)	32 (71.1)	
Medications				
RAAS Inhibitors				
ACEi				0.247^a^
With ACEi	28	14 (50.0)	14 (50.0)	
Without ACEi	80	30 (37.5)	50 (62.5)	
ARB				0.931^a^
With ARB	25	10 (40.0)	15 (60.0)	
Without ARB	83	34 (41.0)	49 (59.0)	
ARNi				0.481^a^
With ARNi	31	11 (35.5)	20 (64.5)	
Without ARNi	77	33 (42.9)	44 (57.1)	
Mineralcorticosteroid antagonist, MRA				0.128^a^
With MRA	61	21 (34.4)	40 (65.6)	
Without MRA	47	23 (48.9)	24 (51.1)	
Loop Diuretics				0.383^a^
With Loop diuretic	57	21 (36.8)	36 (63.2)	
Without Loop diuretic	51	23 (45.1)	28 (54.9)	

* *p*-value of less than 0.05 is considered significant.

a Chi square test.

b Fisher's exact test.

## Discussion

This study provides valuable real-world insights into the safety outcomes of SGLT2 inhibitors and describes changes in clinical parameters following SGLT2 inhibitor initiation in elderly patients with heart failure. It included those with comorbidities such as diabetes, hypertension, chronic kidney disease, and ischemic heart disease, and most were started on empagliflozin, reflecting prescriber preferences. The study encompasses both preserved and reduced ejection fraction types, highlighting the heterogeneity of heart failure among the elderly and enhancing external validity. The cohort was predominantly male, with Malay patients representing the largest ethnic group.

In our cohort, empagliflozin accounted for 96.0% of SGLT2 inhibitor prescriptions (*n* = 121), while dapagliflozin was used in only five patients (4.0%). This reflected local formulary availability and prescribing practices. Although landmark trials evaluated empagliflozin at 10 mg daily, some patients received 12.5 mg (23.0%, *n* = 29) or 25 mg (33.3%, *n* = 42) (13). These patterns reflected routine practice, where clinicians individualized therapy based on glycaemic needs. The 12.5 mg dose was achieved by halving a 25 mg tablet when appropriate.

The median baseline systolic blood pressure was 138 mmHg (IQR: 121–154), within the recommended range for elderly patients with comorbidities, indicating a balance between antihypertensive treatment and the effects of vascular aging (10). Although medications were used, age-related vascular stiffening, impaired baroreceptor sensitivity, and increased arterial resistance persisted, confirming that the cohort reflects typical elderly patients with heart failure.

AKI emerged as the most prevalent adverse event in this study, with an incidence of 12.1%. This incidence was comparable to that reported in the SOLD study, which documented treatment discontinuation due to AKI in 12.5% of 739 elderly patients with diabetes after 12 months (12). Although our cohort was smaller and included elderly patients with heart failure rather than diabetes alone, the similarity in AKI incidence provides supportive real-world context. Importantly, the SOLD study also demonstrated that discontinuations due to adverse events were almost twice as high in patients aged ≥80 years compared with younger groups, underscoring the heightened vulnerability of the very elderly.

Interpreting our 12.1% incidence rate requires consideration of the limitations inherent to our retrospective registry, where detailed clinical data like severity grading, onset timing, and specific treatments weren't consistently recorded. In elderly real-world practice, AKI diagnosis often reflects serum creatinine fluctuations due to volume status or diuretics rather than persistent structural kidney injury. Due to reduced renal reserve and the high prevalence of chronic kidney disease (31.0%) in this cohort, these functional changes may have been observed more frequently, partly accounting for the higher AKI incidence compared with controlled clinical trials.

A meta-analysis demonstrated that SGLT2 inhibitors reduce the risk of AKI, with stronger protection in elderly patients aged 75 years and older. Nevertheless, Gill et al. (2019) reported that AKI may still occur and, in certain cases, necessitate the discontinuation of treatment (20, 21). Taken together, these findings suggest that while SGLT2 inhibitors are generally safe, AKI remains a clinically relevant concern in elderly patients and warrants close renal monitoring.

Other adverse events, including orthostatic hypotension and volume depletion, were uncommon, in contrast to the higher rates reported in the DECLARE–TIMI 58 and SOLD studies (12, 22). This difference may reflect the relatively small sample size and retrospective design of our study. Preventive measures, such as maintaining adequate hydration and optimizing antihypertensive therapy, may help reduce the risk of orthostatic hypotension. UTIs occurred less frequently in our study than those reported by Aldafas et al. and Rigato et al., which may also reflect the relatively small sample size and retrospective design of our study. Hypoglycemia and diabetic ketoacidosis were likewise uncommon, consistent with findings from meta-analyses demonstrating no increased risk. The single event classified as “other” represented an isolated episode of hyperkalaemia, affecting one patient (0.8%). Overall, continued clinical vigilance remains essential given the advanced age and frailty of this population (11, 20).

In this cohort, 40.7% (*n* = 44/108) of elderly patients demonstrated preservation or improvement in eGFR at 12 months. Landmark trials show SGLT2 inhibitors cause an initial, transient eGFR dip due to tubuloglomerular feedback and vasoconstriction, but our 12-month data highlights subsequent stabilization and rebound. Baseline SBP ≤140 mmHg was significantly associated with long-term eGFR improvement (*p* = 0.034), which may reflect the combined benefits of optimized blood pressure control and the favorable hemodynamic effects of SGLT2 inhibitors. The observed renal benefits may be partly explained by the established hemodynamic effects of SGLT2 inhibitors, including improved cardiac function and reduced cardiorenal congestion, which may enhance renal perfusion following the initial eGFR decline. This is consistent with long-term renal preservation reported in EMPEROR-Reduced and real-world elderly populations (13, 19).

Interpretation of the functional efficacy outcomes should consider the baseline NYHA class distribution, with 72.2% of patients classified as NYHA Class I, 24.6% as Class II, and 3.2% as Class III. This distribution reflects routine outpatient practice, where clinically stable elderly patients receiving long-term therapy generally present with mild symptoms. In contrast, those with advanced heart failure are more commonly managed in hospital or specialized heart failure services. Consequently, the predominance of NYHA Class I patients limited the potential for further functional improvement, and the modest improvements observed should therefore be interpreted within this context rather than as evidence of limited therapeutic benefit. Because only a small proportion of patients had advanced symptoms, our findings are primarily applicable to clinically stable elderly outpatients. Future studies including a broader spectrum of NYHA functional classes are warranted to evaluate better the safety and efficacy of SGLT2 inhibitors in patients with more advanced heart failure.

In our cohort, more than half of the patients with available echocardiographic follow-up demonstrated improvement in LVEF, supporting the potential benefits of SGLT2 inhibitors on cardiac function in elderly patients with heart failure. These findings are consistent with previous studies, including Filippatos et al., which reported improvements in LVEF following SGLT2 inhibitor therapy in patients with HFrEF (23). Although the precise mechanisms were not evaluated in our study, the beneficial effects of SGLT2 inhibitors have been attributed to improvements in myocardial energetics, natriuresis, and favorable cardiac remodeling (13).

While SGLT2 inhibitors have demonstrated cardiorenal benefits across the spectrum of heart failure, the magnitude and nature of these benefits vary according to left ventricular ejection fraction (LVEF) phenotype. Landmark trials such as EMPEROR-Reduced and DAPA-HF demonstrated significant reductions in cardiovascular mortality and heart failure hospitalizations among patients with HFrEF. In contrast, EMPEROR-Preserved and DELIVER showed that, in patients with HFpEF and HFmrEF, the principal benefit was a reduction in heart failure hospitalizations, with less pronounced effects on cardiovascular mortality (13, 24–26).

In the present cohort, baseline LVEF was available for 72 patients, with a median baseline LVEF of 37.0% (IQR: 30.0%–46.5%), suggesting that reduced ejection fraction was common within the study population. A key limitation of pooling efficacy outcomes across a heterogeneous heart failure cohort is that changes in LVEF are not uniformly interpretable across heart failure phenotypes. Patients with HFrEF have greater potential for measurable improvement in LVEF following reverse ventricular remodeling. In contrast, patients with HFpEF typically have preserved baseline ejection fractions and may derive meaningful clinical benefit without substantial changes in LVEF. Because subdividing the available sample with baseline LVEF data (*n* = 72) into HFrEF, HFmrEF, and HFpEF subgroups would have resulted in relatively small patient numbers within each category, phenotype-specific subgroup analyses were not performed, as they would have been underpowered and unlikely to provide reliable estimates. Consequently, our findings should be interpreted as overall real-world clinical associations rather than phenotype-specific treatment effects. Future prospective studies with larger, phenotype-stratified cohorts are warranted to evaluate the differential safety and clinical outcomes of SGLT2 inhibitors across individual heart failure phenotypes.

Our study is among the few Malaysian investigations assessing both the safety and efficacy of SGLT2 inhibitors in elderly patients with heart failure, including both HFrEF and HFpEF. Conducted at two hospitals with a heterogeneous patient population, the findings offer clinically relevant insights for routine practice. Multiple clinical endpoints were assessed, providing a comprehensive evaluation of both safety and efficacy outcomes. The therapy was generally well tolerated, with a low rate of adverse events. Notably, improved renal outcomes were observed in patients with well-controlled blood pressure, whereas those with CKD were more prone to AKI, highlighting the need for individualized monitoring and management.

Future research should expand upon this preliminary study by implementing a prospective study with larger cohorts, comprehensive data collection, and extended follow-up periods. Stratifying patients based on frailty, CKD stage, diabetes status, and SGLT2 inhibitor type could elucidate differential risks and benefits. The use of standardized follow-up protocols and the inclusion of functional and quality-of-life assessments would also enhance the outcomes. Overall, these findings support the broader implementation of SGLT2 inhibitors in elderly patients with heart failure while highlighting the value of national registries to monitor long-term safety and effectiveness in routine clinical practice.

Beyond their established benefits on heart failure and renal outcomes, emerging evidence suggests that SGLT2 inhibitors may also confer anti-arrhythmic benefits. A recent systematic review and meta-analysis of adjudicated randomized controlled trials reported that treatment with empagliflozin or dapagliflozin was associated with a significant reduction in the risk of sudden cardiac death compared with placebo, thereby expanding the potential cardiovascular benefit profile of SGLT2 inhibitors beyond heart failure hospitalization and renal protection (27). However, our retrospective electronic health information system (e-HIS) registry did not capture arrhythmia-related outcomes, including sudden cardiac death, ventricular arrhythmias, implantable cardioverter-defibrillator therapies, cardiac arrest, or arrhythmia recurrence. Therefore, our findings cannot provide insight into the potential anti-arrhythmic effects of SGLT2 inhibitors. Future prospective studies incorporating adjudicated arrhythmic endpoints are warranted to further evaluate these potential benefits in elderly patients with heart failure.

In addition to ventricular arrhythmias and sudden cardiac death, atrial fibrillation (AF) is another important cardiovascular comorbidity in elderly patients with heart failure. Emerging evidence suggests that SGLT2 inhibitors may reduce AF burden and recurrence, potentially influencing rhythm-control strategies, including catheter ablation (28). Furthermore, a recent systematic review and meta-analysis demonstrated that, among carefully selected patients undergoing successful AF ablation, individualized oral anticoagulation strategies may reduce major bleeding without compromising thromboembolic outcomes. However, our study did not collect data on AF, catheter ablation, rhythm-control strategies, anticoagulant therapy, or bleeding outcomes. Therefore, these findings are presented solely to provide clinical context and should not be interpreted in relation to our study results. Future prospective studies are warranted to investigate whether the potential reduction in AF recurrence associated with SGLT2 inhibitor therapy may influence catheter ablation outcomes and individualized anticoagulation strategies in elderly patients with heart failure.

Despite its strengths, this study has several important limitations. Most notably, its single-arm retrospective design without a concurrent control group precludes definitive attribution of the observed improvements in LVEF, eGFR, and NYHA class to SGLT2 inhibitor therapyas these outcomes may also have been influenced by the natural course of heart failure, optimization of guideline-directed medical therapy, and lifestyle modifications. Furthermore, the modest sample size limited statistical power, precluded multivariable regression analyses, and restricted adjustment for potential confounding factors. Consequently, the findings should be regarded as exploratory and hypothesis-generating rather than evidence of a causal treatment effect.

The predominance of patients with NYHA Class I at baseline limits the generalizability of our findings to elderly patients with more advanced symptomatic heart failure and may have underestimated the magnitude of functional improvement. In addition, because only 4.0% of patients received dapagliflozin, the findings primarily reflect real-world experience with empagliflozin and should not be interpreted as demonstrating equivalent safety or efficacy for dapagliflozin.

Finally, the retrospective design is inherently susceptible to selection and information bias due to incomplete documentation. Data on medication adherence, actual SGLT2 inhibitor exposure duration, dose modifications, frailty status, and quality of life were not consistently available, limiting a comprehensive assessment of factors influencing treatment outcomes. Future prospective studies with larger cohorts, standardized follow-up, and more comprehensive clinical data are warranted to validate these findings and further evaluate the safety and efficacy of SGLT2 inhibitors in elderly patients with heart failure.

## Conclusion

In this retrospective multicenter cohort of elderly patients with heart failure, SGLT2 inhibitor initiation was associated with observed improvements in LVEF (51.4%), renal function (40.7%), NYHA functional class (21.4%), and HbA1c (38.2%). Acute kidney injury was the most frequently recorded adverse event (12.1%), occurring predominantly among patients with pre-existing chronic kidney disease. Although favorable changes in clinical parameters were observed, the absence of an unexposed control group precludes attributing these findings solely to SGLT2 inhibitor therapy. These observational findings emphasize the importance of individualized clinical monitoring and support the need for future prospective controlled studies to evaluate further the safety and clinical outcomes of SGLT2 inhibitors in elderly patients with heart failure.

## Data Availability

The raw data supporting the conclusions of this article will be made available by the authors, without undue reservation.
